# Extragonadal germ cell tumor presenting in a woman with systemic lupus erythematosus: a case report

**DOI:** 10.1186/1752-1947-4-97

**Published:** 2010-03-25

**Authors:** Mohd Shahrir, Abdul Halim, Soehardy Zainudin, Rozita Mohamad, Loo C Yuen, Rashidi Saidin, Norella Kong

**Affiliations:** 1 Department of Medicine, Faculty of Medicine, Universiti Kebangsaan, Malaysia

## Abstract

**Introduction:**

Germ cell tumor of the pituitary gland is a very rare occurrence.

**Case presentation:**

We describe the case of a 28-year-old Malaysian Malay woman with lupus nephritis who complained of a three month headache and blurring of vision. She was found to have a pituitary mass, which was later proven to be a germ cell tumor. As of writing this case report, her disease is in remission.

**Conclusion:**

The disruption of the pituitary gonad axis could affect the disease activity by reducing immunoregulatory control.

## Introduction

Germ cell tumor of the pituitary gland is a very rare occurrence. This case report describes a 28-year-old Malaysian Malay woman with lupus nephritis who complained of headache and blurring of vision. She was later found to have a pituitary germ cell tumor.

Pituitary germ cell tumor is considered as a type of extragonadal germ cell tumor. They represent 5% of germ cell tumors and typically arise in midline locations. The specific location of the tumor varies with the patient's age [1]. The most common sites of origin in adults are the anterior mediastinum, the retroperitoneum, and the pineal and suprasellar regions of the brain. Meanwhile, in infants and young children, the sacrococcyx is the most common site of extragonadal germ cell tumors, followed by intracranial sites [2]. In contrast to primary gonadal germ cell tumors, the only known risk factor for extragonadal germ cell tumors is the Klinefelter syndrome (47XXY), which is associated with mediastinal nonseminomatous germ cell tumors [3].

The age of onset, site of origin, and histologic type of the tumor are important distinguishing characteristics for its prognosis and treatment.

## Case presentation

A 28-year-old Malaysian Malay woman presented in 1994 with musculoskeletal manifestation of systemic lupus erythematosus (SLE). In 1999, she developed nephrotic syndrome secondary to lupus nephritis (World Health Organization Class III). She was immediately started on low-dose cyclophosphamide and mycophenolate mofetil treatment.

A year later, however, she complained of amenorrhoea. Levels of follicle-stimulating hormone and luteinizing hormone were both lower than would have been expected. Nine months later, she complained of persistent headaches and blurring of vision. She was screened for chronic meningitis but had a normal brain computed tomography (CT) scan. However, we were not able to do a lumbar puncture as she refused this procedure. Her SLE was already in remission at that time.

On examination, both her peri-orbital areas were swollen and red. Her conjunctivae were also injected. She had bilateral temporal hemianopia with left optic atrophy. Magnetic resonance imaging revealed a lobulated mass which extended into the suprasellar cistern and floor of her third ventricle, splaying the optic chiasm.

**Figure 1:**
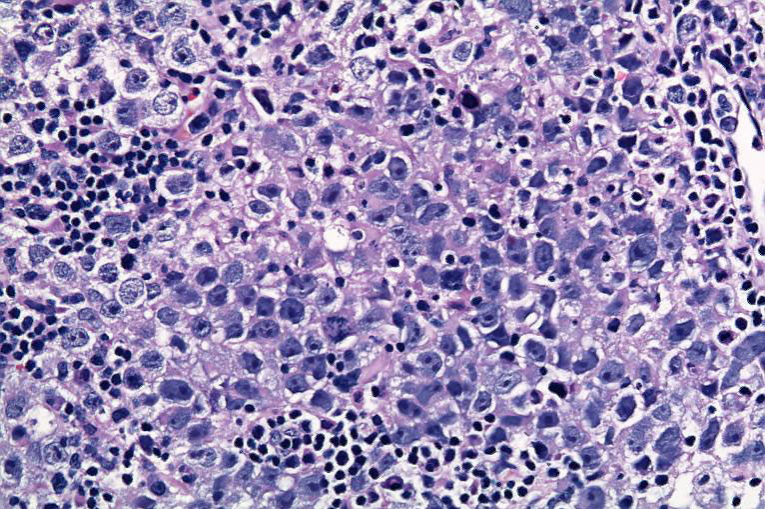
**An image of our patient's pituitary showing polymorphic cells with granular cytoplasm**.

She underwent pituitary resection and her histopathological examination later revealed a germ cell tumor (Figure 1). She was referred subsequently for radiotherapy.

## Discussion

This case study is unusual because our patient had both SLE and pituitary germ cell tumor. It is also unusual because when our patient's pituitary function was disrupted, her lupus nephritis went into remission. Initially, it was thought that the two mechanisms responsible for her amenorrhoea were gonadal injury secondary to SLE insult and cyclophosphamide usage.

However, our patient's amenorrhoea was due to the pituitary involvement and its effect on her ovaries. At the same time, her SLE was also in remission. This can be explained by the fact that the reduction in immunoregulatory functions of estradiol, testosterone, progesterone, dehydroepiandrosterone (DHEA) and prolactin had resulted in the remission of our patient's SLE [4].

## Conclusion

The functional disruption of the pituitary-gonadal complex could affect SLE activity by reducing our patient's hormonal immunoregulation.

## Consent

Written informed consent was obtained from our patient for publication of this case report and any accompanying images. A copy of the written consent is available for review by the Editor-in-Chief of this journal.

## Competing interests

The authors declare that they have no competing interests.

## Authors' contributions

All the authors contributed to the management of the patient. Additionally, MS was the primary author of the manuscript. All authors read and approved the final manuscript.
